# Alginate-Induced Disease Resistance in Plants

**DOI:** 10.3390/polym14040661

**Published:** 2022-02-09

**Authors:** Roohallah Saberi Riseh, Mozhgan Gholizadeh Vazvani, Marzieh Ebrahimi-Zarandi, Yury A. Skorik

**Affiliations:** 1 Department of Plant Protection, Faculty of Agriculture, Vali-e-Asr University of Rafsanjan, Imam Khomeini Square, Rafsanjan 7718897111, Iran; r.saberi@vru.ac.ir (R.S.R.); mgholizadehvazvani@yahoo.com (M.G.V.); 2 Department of Plant Protection, Faculty of Agriculture, Shahid Bahonar University of Kerman, Kerman 7618411764, Iran; ebrahimimarzieh@gmail.com; 3 Institute of Macromolecular Compounds of the Russian Academy of Sciences, Bolshoi VO 31, 199004 St. Petersburg, Russia

**Keywords:** induced disease resistance, sodium alginate, polysaccharides, plant growth-promoting bacteria

## Abstract

Plants are continuously exposed to a wide range of pathogens, including fungi, bacteria, nematodes, and viruses; therefore, survival under these conditions requires a sophisticated defense system. The activation of defense responses and related signals in plants is regulated mainly by the hormones salicylic acid, jasmonic acid, and ethylene. Resistance to pathogen infection can be induced in plants by various biotic and abiotic agents. For many years, the use of abiotic plant resistance inducers has been considered in integrated disease management programs. Recently, natural inducer compounds, such as alginates, have become a focus of interest due to their environmentally friendly nature and their ability to stimulate plant defense mechanisms and enhance growth. Polysaccharides and the oligosaccharides derived from them are examples of eco-compatible compounds that can enhance plant growth while also inducing plant resistance against pathogens and triggering the expression of the salicylic acid-dependent defense pathway.

## 1. Introduction

**Figure 1:**
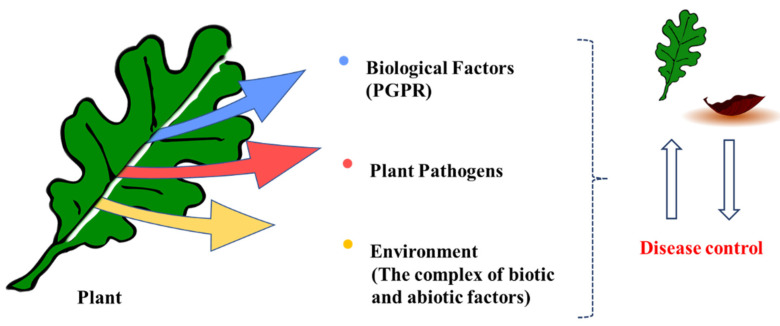
Complex interactions between the plant, the pathogen, plant growth-promoting rhizobacteria as biological factors (inducers), and the environment.

Plant pathogens cause diseases with different pathogenicity mechanisms in various parts of plants, resulting in great economic loss [1]. Pathogens successfully infect plants through mechanisms involving the recognition of plant surface receptors, production of virulence and effector proteins, and overcoming plant defense barriers [1,2]. During their evolution, pathogens maintain their attacks on plants by emerging new races, while plants develop mechanisms to cope with and adapt to these new pathogen variants. When a pathogen attacks a plant, it also triggers signal pathways that elicit the expression of the plant’s defense genes [3], which activate defense responses against the pathogen [4]. As soon as the plant initiates this immune response, the pathogen contamination process is disrupted, and its gene expression is suppressed. These defense responses can be activated by both endogenous (plant structure) and exogenous (biotic and abiotic) elicitors [5]. Exogenous agents are now broadly used in agriculture worldwide to control the losses caused by different pathogens. The use of genetic resistance to pathogens, the identification of resistance genes, and the transfer of these genes to plants require long-term breeding and genetic engineering programs. By contrast, due to the complex interaction that occurs between the pathogen, plant, biological inducer components, and the environment (combined biotic and abiotic factors), the application of biotic inducers to control plant diseases in field conditions may lead to changes in the mechanism of induction of resistance and the form of the defense signals (Figure 1). The interactions among these three biotic agents (plants, pathogens, and biological inducers) and with the environment must, therefore, be controlled to achieve maximum disease control. Plants, pathogens, and biological factors may respond to different environmental conditions through changes that may affect the mechanism of resistance. Unfortunately, the consequence of the complex quadruple effects (pathogen × plant × inducer agents × environment) that occur between these factors is a less successful biological control in the field, compared with the control achieved in the controlled environments of the greenhouse and laboratory.

The role of abiotic agents in the induction of resistance against phytopathogens has been reported in many studies [6,7,8]. One example is β-aminobutyric acid, which has been successfully exploited in practical agriculture for defense priming in different crops [9]. Many natural compounds have now been demonstrated to enhance the defense priming response in plants, ranging from oligosaccharides, glycosides, and amides to vitamins, carboxylic acids, and aromatic compounds [10]. A simple compound, hexanoic acid, shows a potent natural priming capability to protect plants against a wide range of pathogens by inducing callose deposition and activating salicylic acid (SA) and jasmonic acid (JA) pathways [7]. Zhou et al. [11] reported that thiamine (vitamin B1) can modulate cellular redox status to protect Arabidopsis against infection by *Sclerotinia sclerotiorum*. Para-aminobenzoic acid, another member of the vitamin B group, was able to enhance resistance against the cucumber mosaic virus and *Xanthomonas axonopodis* by inducing systemic acquired resistance (SAR). In the same study, benzothiadiazole was also shown to reduce disease severity, but it also caused adverse effects on the plant, as shoot lengths were shortened and cucumber fruit lengths were significantly reduced, compared with plants treated with para-aminobenzoic acid or untreated control plants [12]. Chitosan, a deacetylated derivative of chitin, can enhance plant defenses by various mechanisms, including scavenging reactive oxygen species (ROS), upregulating antioxidant activities, and activating the octadecanoid pathway that leads to the production of phytoprotective fatty acids [13].

The oligosaccharide products arising from the hydrolysis of natural polysaccharides can also serve as elicitors that induce resistance and alter the expression of plant defense genes [4]. For example, exogenous application of oligogalacturonide can induce plant defense responses, such as accumulation of phytoalexin, β-1,3-glucanase, and chitinase, and generation of ROS, by triggering nitric oxide (NO) production [14]. Oligochitosan can protect plants against fungi, bacteria, and viruses by activating the SA and jasmonic acid–ethylene (JA–ET) pathways, while also protecting against abiotic stresses by the induction of an abscisic acid (ABA)-dependent pathway [15]. Microbial products can also induce defense responses in plants; an example is an *Agrobacterium* spp. fermentation product, oligocurdlan, which has been shown to induce defense responses against *Phytophthora infestans* in potatoes [16]. Other examples are the oligosaccharides that naturally occur in green and brown algae and that can activate defense signals in plants [17,18]. These compounds are also used as fertilizers and soil conditioners in agricultural and horticultural industries [19]. 

Several commercial products containing oligosaccharides are now successfully marketed for plant protection. One example is FytoSave^®^ (LIDA Plant Research, Valencia, Spain), a complex mixture of oligochitosans and oligopectates that is active against downy mildew infection in grape and cucumber [20]. The active component of FytoSave^®^ (LIDA Plant Research, Valencia, Spain), COS–OGA, can induce resistance against *Phytophthora infestans,* the causal agent of potato late blight, by enhancing pathogenesis-related (PR) proteins, such as PR-1 and PR-2. The induction of resistance in plants by COS–OGA is reported as a cumulative process involving SA. COS–OGA combines cationic chitosan oligomers, chitooligosaccharides (COSs), with anionic pectin oligomers, oligogalacturonides (OGAs) [20,21]. In 2018, FytoSave^®^ product (LIDA Plant Research, Valencia, Spain) as the first plant phytovaccine with phytosanitary registration was admitted by the European Commission for use in organic agriculture (https://www.infoagro.com, accessed on 8 January 2022). Another commercial product is Stemicol^®^ (LIDA Plant Research, Valencia, Spain), a mixture of chitooligosaccharides that causes the reduction in fruit rot in tomatoes, strawberries, and grapes (https://www.lidaplantresearch.com/phytovaccines/stemicol, accessed on 8 January 2022). Thus, natural compounds, such as oligosaccharides, are now promising alternatives to chemical fungicides for controlling pathogen diseases in the field [22].

Another plant defense elicitor of considerable interest is sodium alginate (ALG), a polysaccharide derived from seaweeds. ALG oligosaccharides or oligoalginates (AOS) are recognized as new types of functional material and are used to enhance seed germination, shoot elongation, root growth, and resistance against plant pathogens [23,24,25,26,27]. AOS can activate the production of phosphodiesterase in suspension cultures of plant cells by modulating the production of ROS and by activating PR proteins and defense enzymes, such as peroxidase (POD), catalase (CAT), polyphenol oxidase (PPO), and phenylalanine ammonia-lyase (PAL) [28]. 

Induced resistance is a suitable alternative strategy for chemical pesticides to control plant diseases. Finding new natural sources of elicitors and exploring their effects on plant defense is a significant issue. Recently, natural inducer compounds, such as ALG, have become a focus of interest due to their environmentally friendly nature and their ability to stimulate plant defense mechanisms and enhance growth. In this review, we discuss the main defense pathways invoked by plants to combat pathogen attacks, with a more intense focus on the role of ALG and AOS in the induction of resistance against plant diseases.

## 2. Plant Immune System against Pathogens

Plant cells are capable of sensing evolutionarily conserved microbial molecular signals, termed pathogen-associated or microbe-associated molecular patterns (PAMPs or MAMPs), through plant pattern recognition receptors [29,30,31]. The PAMP molecules are essential for pathogen fitness; therefore, they represent an efficient form that plants exploit to sense the presence of pathogens. The perception of PAMPs by plant pattern recognition receptors activates an immune response, referred to as PAMP-triggered immunity, which provides protection against nonhost pathogens and limits diseases caused by virulent pathogens [32].

However, pathogens also adapt to their host plants and evolve mechanisms for the suppression of plant defenses induced by pathogenicity signals and genes [33,34,35,36]. In return, plants evolve resistance proteins (R proteins) that can detect, either directly or indirectly, the effector proteins of the pathogen and trigger a different form of disease resistance, known as effector-triggered immunity, which is highly specific and often accompanied by the appearance of the hypersensitive response (HR) and SAR in the plant. Damage-associated molecular patterns, which include plant cell walls and cutin fragments characteristically released by the enzymatic actions of pathogens, can serve as triggers of immune responses in plants [31,37,38]. The effector-triggered immunity and PAMP-triggered immunity pathways activate a set of downstream defense responses, including signaling pathways and transcription factors that limit pathogen proliferation or disease symptom expression [39]. Further, ROS accumulate, cell wall defense mechanisms are activated, and defense hormones such as SA, ET, and JA accumulate. Crosstalk between the SA and JA–ET signaling pathways has also emerged as an important regulatory mechanism in plant immunity [32,40,41,42,43].

Plants are equipped with various defense genes, but the expression of these genes is often latent in healthy conditions. Intriguingly, these defense genes can be induced in plants by the application of any type of inducer in a process known as induced resistance [44]. The inducer triggers the plant‘s defense system against a subsequent pathogen attack, thereby suppressing the occurrence of disease. Induced resistance activates a wide range of defense mechanisms, and the defense signals in this pathway lead to two types of resistance: SAR and induced systemic resistance (ISR) [45]. 

### 2.1. Systemic Acquired Resistance (SAR)

SAR describes a type of plant defense response that provides long-term protection against various plant pathogens. The systemic signals involved in SAR include SA, lipid-based signal molecules, and ROS; these molecules transport the systemic signal that is activated by the plant–pathogen interaction [46]. SAR is related to the production of SA as a signaling molecule and the accumulation of PR proteins [46]. SAR can be activated in many plant species by different pathogens that cause necrosis or hypersensitive reactions in plants. This type of resistance is long-lasting and effective against a broad spectrum of pathogens [47,48]. SA is a defense hormone, and pathogen infections induce SA synthesis by upregulating the expression of isochorismate synthase 1 (*ICS1*), a gene that encodes a key enzyme in the SA synthesis pathway [49]. The enhancement of another defense signal for SAR—namely, the increased expression of palmitic acid and its derivatives, has been observed in the primed guard cells of Arabidopsis plants [50]. NO and ROS, which are both early chemical signals in systemic immunity, operate in a feedback loop in SAR. ROS also act additively to mediate the chemical hydrolysis of unsaturated fatty acids to induce SAR in plants [51]. During SAR, SA binds the H_2_O_2_-scavenging enzymes, CAT, and ascorbate peroxidase, and inhibits their activities, thereby promoting an increase in H_2_O_2_ levels. This increase is then responsible for the signal transduction that leads to the induction of pathogenesis-related genes and pathogen resistance [46].

### 2.2. Induced Systemic Resistance (ISR)

Plant growth-promoting rhizobacteria (PGPR) colonize the root surface, thus preventing the penetration of pathogens while inducing systemic resistance in plants. A specific recognition response is needed between the plant and the rhizobacteria for the onset of ISR [52]. Rhizobacterial determinants, such as flagellar proteins, lipopolysaccharides, antibiotics, quorum-sensing molecules, volatile organic compounds, and siderophores, can elicit ISR [53,54]. When this type of resistance occurs, the plant’s immune system is strengthened against other invaders [55].

ISR is a nonspecific response, as indicated by its broad action against different pathogens [56]. ISR is generally activated by a pathway in which JA and ET are central players [57]. Although beneficial rhizobacteria often trigger JA–ET-dependent pathways, several PGPR have been reported to trigger SA-dependent pathways [58]. Some of the signal pathways that regulate ISR are similar to those of SAR [45,57,58]. One example is NPR1, a common regulator of both SAR and ISR pathways that functions as a transcriptional coactivator of SA-responsive pathogenesis-related genes. However, the role of NPR1 in ISR has not yet been established [58,59].

Immune responses are induced in plants by many biological and chemical stimuli that trigger defense priming and increase the plant’s defense capacity. Priming is defined as enhanced sensitivity and responsiveness to stress that results from prior experience and leads to increased resistance. Primed plants respond faster and have stronger defense responses against subsequent stresses [60]. Table 1 shows examples of biological priming agents and the mechanisms by which they induce resistance against pathogens in different plants.

## 3. Abiotic Inducers of Disease Resistance in Plants

Abiotic inducers include chemicals that act at various points in the signaling pathways involved in disease resistance and against biotic and abiotic stress. One compound, 2,6-dichloroisonicotinic acid, and its methyl ester were the first synthetic compounds shown to prime defense responses in plants [73]. A wide range of cellular responses, including alterations in ion transport across the plasma membrane, synthesis of antimicrobial secondary metabolites (e.g., phytoalexins, cell wall phenolics, and lignin-like polymers), and activation of defense genes, are potentiated by these chemical inducers [6]. The resistance induced by chemical elicitors is broad spectrum and long-lasting, and many of these elicitors provide disease control ranging between 20% and 85% [74]. For instance, exposure of plants to β-aminobutyric acid, probenazole, benzothiadiazole, and SA can all induce resistance against a broad range of pathogens [75]. Durable induced resistance, based on priming of gene expression, was reported after treatment of tomato seeds with β-aminobutyric acid or JA [76]. Similarly, the treatment of faba beans with acibenzolar-S-methyl induced SAR against rust and ascochyta blight diseases in both greenhouse and field conditions, and this protection was still evident several weeks after acibenzolar-S-methyl application [77]. Table 2 shows examples of abiotic components known to induce pathogen resistance in plants.

As the world’s population expands, the demand for food production increases. Therefore, agriculture must be able to meet the nutritional needs of people throughout the world, making the protection of crops from plant pests and pathogens paramount. Therefore, new ways appear to be needed to stimulate the defense genes in plants to suppress pathogen attacks. The application of abiotic inducer agents derived from natural factors represents an environmentally friendly way to trigger the induction of resistance in the field. The plant’s defense system is highly triggerable; therefore, the existence of an external abiotic inducer factor that has no adverse effect on the environment can play a major role in activating the plant defense system and suppressing pathogens. 

Environmentally friendly polymer compounds, especially ALG, are compatible compounds that stimulate plant defense mechanisms. The use of these abiotic materials avoids the known toxic effects of synthetic chemical pesticide agents on humans and other nontarget organisms. These compounds are able to induce plant resistance against pathogens and increase the expression of SA-dependent defense pathways. In what follows, we discuss the advantages of polysaccharides and the mechanisms of ALG in the induction of resistance against plant pathogens.

### 3.1. Polysaccharides as Plant Defense Inducers

The plant’s defense system is fundamental to its ability to resist pathogens and is, therefore, an effective target for research on disease management. Plants recognize pathogens using PAMPs with structures or chemical patterns similar to their pathogens [31,87]. Therefore, not surprisingly, oligosaccharides that share structures similar to the components of pathogen cell walls or other structures can also serve as PAMPs to activate the plant immune system [87,88]. 

The promotion of eco-friendly alternatives is necessary to reduce the environmental effects of present-day chemicals used in agriculture [89]. In recent decades, there have been many reports regarding the induction of defense resistance by the application of plant extracts and essential oils, microbial (bacteria, fungi, and microalgae) extracts, seaweed extracts, and polysaccharides. Polysaccharides with high structural complexity and biological activity have become ideal and environmentally friendly biological resources for inducing resistance against plant pathogens [89,90,91,92,93]. The effects of polysaccharides obtained from microalgae and cyanobacteria on the biochemical and metabolomic markers linked to defense pathways in tomato plants were evaluated by Rachidi et al. [89]. The polysaccharides extracted from *Phaeodactylum* *triocnutum*, *Desmodesmus* sp., and *Porphyridium* sp. improved the activities of phenylalanine ammonia-lyase, chitinase, β-1,3 glucanase, and peroxidase enzymes in tomato leaves [89]. Further, GC–MS metabolomics analysis revealed that polysaccharides induced the modification of metabolite profiles, such as fatty acids, alkanes, and phytosterol, in tomato leaves [89].

Pettongkhao et al. [94] reported that sulfated polysaccharide from *Acanthophora spicifera*, a red alga, induced defense responses against *Phytophthora palmivora* in a rubber tree (*Hevea brasiliensis*). Their results showed that the extracted crude polysaccharide induced SA and scopoletin accumulation and SA-responsive gene expression but suppressed JA-responsive gene expression [94]. An elicitor from the green algae *Ulva* spp. caused the protection of *Medicago truncatula* against infection by *Colletotrichum trifolii* [95]. A broad range of defense-related transcripts upregulated notable genes involved in the biosynthesis of phytoalexins, PR proteins, and cell wall proteins [95].

One polysaccharide, tramesan, obtained from *Trametes versicolor*, caused an increase in the JA level and the early expression of plant defense genes against Septoria Leaf Blotch complex disease in wheat [96]. The use of biopolymers as elicitors for controlling plant diseases is gaining momentum worldwide due to the eco-friendly and nontoxic nature of polysaccharides. These materials have the added advantage of being sufficiently resistant to degradation by hydrolytic enzymes and by exposure to acidic environments [97,98].

Oligosaccharides are low molecular weight carbohydrates that arise from the degradation of polysaccharides [15]. These compounds have biological activity in many living organisms [99]. In plants, they regulate specific processes, such as cell morphogenesis and the pH-dependent development of flowers or callus, and in general, they modulate plant growth. The use of oligosaccharides can increase soil fertility and activate plant defense against both biotic and abiotic stresses [15]. 

### 3.2. Alginate and Induction of Resistance against Plant Pathogens

Algal polysaccharides are among the most abundant organic molecules in nature and have great diversity, as well as the potential to induce resistance in plants [27,100,101]. ALG is extracted from the cell walls of brown macroalgae (e.g., *Macrocystis pyrifera*, *Laminaria hyperborean*, *Ascophyllum nodosum*), and several bacteria (*Azotobacter vinelandii*, *Pseudomonas* spp.) contain ALG at up to 40% of their dry weight [102].

**Figure 2:**
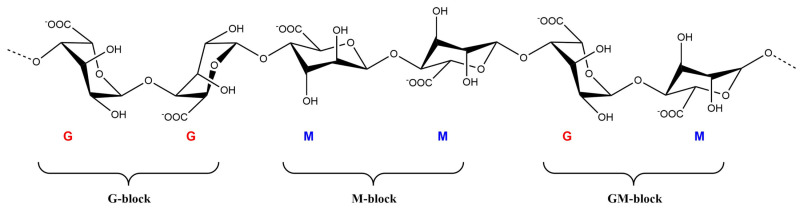
Chemical structure of alginate.

ALG is a linear biopolysaccharide copolymer consisting of 1,4-linked β-D-mannuronate (M) and α-L-guluronate (G), which can be arranged in heteropolymeric and homopolymeric blocks (Figure 2) [27,103]. Due to their hydrophilic properties, ALG hydrogels can absorb large amounts of water or biological fluids without losing their structure. ALG is a nontoxic and environmentally friendly polysaccharide that can be used as a delivery vehicle in various applications due to its unique physicochemical properties [102]. Alginic acid is insoluble in water or organic solvents, but its monovalent alginate salts are soluble in water and organic solvents and form stable solutions in water [102].

**Figure 3:**
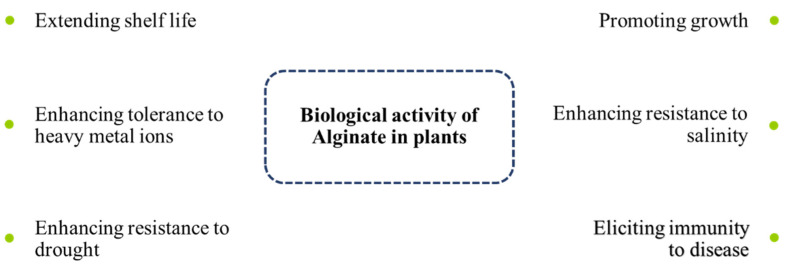
Biological activity of alginate in plants against different stresses.

The linear ALG polymer, at physiological temperature and pH, and in the presence of some chemical initiators, can be converted to a three-dimensional polymer by a process called free-radical polymerization [102,104,105]. During this polymerization, some chemicals can be easily combined into the forming hydrogel to generate a liquid–solid phase under physiological conditions [106,107]. ALG is widely used in this way in medicine to encapsulate various drugs for delivery to target organs and tissues. The formation of hydrogels allows the use of ALG as a carrier of proteins, DNA, and live cells while maintaining their biological activity [108]. ALG is also able to stimulate the growth and development of plants and induce resistance to biotic and abiotic stresses [109]. Phenolic compounds (as secondary metabolites) can cross-link with ALG to strengthen plant cell walls against pathogen attack [110]. Figure 3 shows the biological activity of ALG in plants against different stresses and environmental factors.

ALG has received much attention due to its environmental compatibility and nontoxic properties as an elicitor in the control of plant diseases [27]. In one study, ALG was investigated as a factor in the induction of resistance against *Alternaria solani*, the causal agent of tomato blight disease [27]. Tomato leaves were treated with different concentrations of ALG (0.2, 0.4, and 0.6%) two days before infection with the pathogen. ALG effectively controlled the growth of *A. solani* in the treated tomato plants and significantly enhanced the expression levels of superoxide dismutase (SOD) in response to infection. Staining of infected tomato leaves with Uvitex-2B and observation by fluorescence microscopy showed significant reductions in pathogen colonization following ALG treatment. ALG at a concentration of 0.4% was very effective in controlling fungal hyphal growth. The level of defense enzymes, including SOD, GPX, and CAT, was enhanced in the treated tomato plants [27]. Identification of the induced resistance mechanisms in tomato by ALG against blight disease was further explored by examining the expression changes in defense marker genes, including β-1,3-glucanase (*PR2*), chitinase (*PR4*), nonexpressor of pathogenesis-related protein 1 (*NPR1*; related to SA signaling pathways), 1-aminocyclopropane-1-carboxylate oxidase (*ACO1*; related to ET signaling pathways), and lipoxygenase D (*LoxD*; related to JA signaling pathways). The expression levels of *PR2*, *NPR1, LoxD*, and *ACO1* were significantly upregulated in leaves treated with *A. solani* and 0.4–0.6% ALG [27]. *PR4* expression was upregulated in pathogen-infected leaves when compared with uninfected control leaves and 0.4% or 0.6% ALG-pretreated leaves infected with pathogen [27].

The major cell wall components of many phytopathogenic fungi are chitin and glucan. Therefore, plant β-1,3-glucanases and chitinases play antifungal roles by hydrolyzing the fungal cell wall. Further, β-1,3-glucanases and chitinases exhibit indirect effects via the formation of oligosaccharide elicitors, which further induce the expression of other PR proteins [111]. The ALG-induced defense responses, therefore, arise by activation of antioxidant enzymes and PR proteins against *A. solani*, to inhibit disease development in tomato seedlings [27]. 

Much interest is now expressed in the use of protein elicitors enclosed in a complex with biopolymers, such as ALG, to protect them against adverse external factors, facilitate their interaction with plant cell receptors, and invoke disease resistance [112,113]. Peptidylprolyl isomerases (PPIases) play roles in the folding of synthesized proteins, immune system responses, transcriptional regulation, cell cycle control, and nuclear events [114]. In one study, the FKBP-type PPIase from *Pseudomonas fluorescens*, which has significant eliciting activity regarding a wide range of plant pathogens, was encapsulated in ALG microparticles [26]. Synergistic interaction between ALG and other compounds was promoted by constructing microparticles consisting of 70% ALG, 20% bovine serum albumin (BSA), and 10% PPIase and evaluating three different plant–pathogen models (tobacco–TMV, tobacco–*A. longipes*, and wheat–*Stagonospora nodorum*). In the wheat–*S. nodorum* model system, a significant eliciting activity of the ALG–albumin complex was observed, and the activity of encapsulated PPIase increased, compared with the free PPIase. The ALG–BSA complex had an eliciting activity that suppressed the development of *A. longipes* on tobacco plants. The PPIase ALG biopolymer complex served as an antipathogenic compound and an inducer of resistance against pathogens in a wide range of plants while also helping to promote plant growth [26]. In the TMV–tobacco model system, no significant differences were observed between PPIase and ALG–BSA–PPIase, and in these treatments, the average amount of necroses per leaf decreased 32–35 times. compared with the control. No eliciting activity was revealed in the case of ALG–BSA [26].

The role of AOS in the induction of resistance against *Pseudomonas syringae* pv. *tomato* DC3000 was evaluated in Arabidopsis by Zhang et al. [25]. Arabidopsis were pretreated by spraying with different concentrations of AOS (25, 50, 100, and 200 mg/L) three days before inoculation with *P. syringae* pv. *tomato* DC3000. The disease index, bacterial growth, production of ROS, and qualitative and quantitative detection of NO and SA were then evaluated. The qRT–PCR analysis revealed an increase in induced immunity against this disease in Arabidopsis. The expression of the *avrPtoB* gene, which represents the pathogenic mechanism of this bacterium, was significantly reduced in leaves treated with AOS, compared with the control leaves. AOS also prevented the growth of bacteria on the leaves. At 25 mg/L, AOS induced both NO and ROS production against the pathogen in Arabidopsis. ROS and NO are the primary signals that initiate defense reactions against plant pathogens [115,116,117,118,119]. After pretreatment with AOS, the SA pathway was activated and significantly enhanced *PR1* expression [25].

Zhang et al. [4] also investigated the activity of AOS and its potential application for the protection of rice plants against *Magnaporthe grisea*. Germinating rice seeds were detached from 5–7-day-old seedlings when the sprouts were 1–2 cm in length and then were treated with AOS. The AOS activity on germinating rice was assayed by determining the accumulation of phytoalexin in seed tissues as a marker of plant disease resistance. The activities of PAL, CAT, and POD were determined in the treated leaves of rice with AOS. An enhancement in PAL activity was detected in the rice leaves treated with AOS. PAL activity is considered to represent a direct response of the host plant to suppress a pathogen attack and is associated with disease resistance. This enzyme was induced by the application of exogenous elicitors, such as abiotic inducer agents [4,120]. CAT, POD, and PAL have a synergistic role in plant disease protection. The production of four kinds of phytoalexin—oryzalexin A, oryzalexin C, phytocassane A/D, and phytocassane B/C—was elicited in rice-seed tissues by AOS. The accumulation of oryzalexin C could be considered a more sensitive marker for assaying elicitor activity [4].

In another study, ALG isolated from the brown seaweed *Bifurcaria bifurcata* and AOS were evaluated for their ability to stimulate the natural defenses of tomato seedlings [121]. PAL activity and polyphenol levels were measured in leaves treated with ALG [121]. PAL activity increased 12 h after treatment. Polysaccharides extracted from *B. bifurcata* and the oligosaccharide derivatives of those polysaccharides significantly induced phenylpropanoid metabolism in tomato seedlings. ALG and its oligosaccharide derivatives should, therefore, be considered potential bioresources for plant protection against phytopathogens in the context of eco-sustainable green technology [121].

Other studies have confirmed that an ALG−lentinan−aminooligosaccharide hydrogel induces strong plant resistance against TMV and increases the release of calcium ions to promote the growth of *Nicotiana benthamiana* [122]. Table 3 shows other studies on the role of ALG in the induction of resistance against plant diseases. 

Based on the studies mentioned above, ALG and AOS are effective elicitors for inducing resistance in plants against various pathogens including fungi, bacteria, and viruses. Both the SA and JA–ET pathways are triggered by these elicitors, and there is evidence of ABA-dependent pathway activation by AOS [15,129]. Therefore, AOS can induce resistance to abiotic stress, such as drought, salinity, and heavy metals, by triggering the ABA signaling pathway in plants [129,130,131]. 

**Figure 4:**
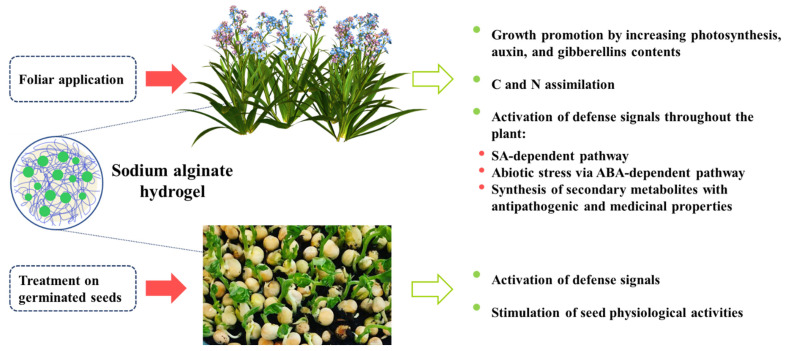
The mechanism of induction of plant disease resistance by alginate.

Figure 4 shows a scheme for seed treatment with ALG, pathogen attack, and the defense pathways that are activated.

## 4. Conclusions

Resistance to plant diseases is a very important issue that should be given great attention. Some plant genotypes and cultivars have a natural resistance to plant pests and diseases. Some have a protective wax-like layer on their surface that prevents damage from pathogens. Others respond to the presence of factors that stimulate the plant’s immune system as an effective way to promote resistance to disease. However, the introduction of resistant cultivars and gene transfer to nonresistant cultivars is an extensive plant breeding process. Further, the geographic compatibility of the introduced resistant cultivars must be considered. Biological control agents, such as beneficial bacteria and nonpathogenic strains, have led to the successful control of many pathogens in the greenhouse and laboratory. However, these agents may fail under field conditions due to complex interactions between the environment, pathogens, plants, and biological factors (e.g., PGPR). Therefore, abiotic inducer compounds that are environmentally friendly and can trigger plant resistance under adverse conditions are very important candidates for research on plant disease resistance. 

ALG is a natural polymer that, due to its potential properties, has been considered a viable choice for the induction of plant resistance against pathogens. This polymeric compound plays a role by stimulating plant defense signals and activating defense genes. Treatment of plants with this compound leads to the activation of SA and JA pathways that protect against pathogen attacks. Plant defense responses, such as the synthesis of phenolic compounds, lignin, PPO, PAL, and PR proteins, are significantly increased in plants treated with ALG, and these responses induce disease resistance. Extensive applications of ALG in the field confirm its effects on the activation of SAR and ISR against a wide range of pathogens. However, induced resistance is a host response and can be influenced in practice by factors such as plant genotype, crop nutrition, frequency, and the method of elicitor application under field conditions.

## Figures and Tables

**Table 1:** Examples of biological agents that induce resistance against plant pathogens.

Biological Inducer	Pathogens	Host	Mechanism	Reference
*Pseudomonas* spp.	*Botrytis cinerea*	Grapevine	Oxidative burst and phytoalexin accumulation in grape cells and leaves.	[61]
	*Clavibacter michiganensis*	Tomato	Increase in levels of PR1a and ACO transcripts and SA signaling pathways.	[62]
	*Meloidogyne* spp.	Tomato	SA production by bacteria.	[63]
	*Pythium aphanidermatum*	Cucumber	Reduced pathogen spread.	[64]
*Bacillus* spp.	*Heterodera glycines*	Soybean	Expression of defense-related genes involved in the SA and JA pathways.	[65]
	*Fusarium* sp.	Tomato	Production of phthalic acid methyl ester by Bacillus.	[66]
	*Botrytis cinerea*	Arabidopsis	Activation of the JA–ET signaling pathway.	[67]
*Trichoderma* spp.	*Botrytis cinerea*	Tomato	Activation of the JA, SA, and ABA signaling pathways.	[68]
			Enhanced activation of jasmonate-responsive genes.	[69]
	*Sclerotinia sclerotiorum*	*Brassica napus*	Induction of SA- and JA–ET-dependent defenses and decreased disease symptoms.	[70]
Mycorrhizal fungi	*Botrytis cinerea*	Lettuce	Provision of biotic stress protection with no nutritional or growth benefits.	[71]
	*Blumeria graminis* f.sp. *tritici*	Wheat	Accumulation of phenolic compounds and H_2_O_2_, upregulation of genes encoding several defense markers (POD, PAL, chitinase 1)	[72]

**Table 2:** Abiotic components that induce pathogen resistance in plants.

Abiotic Component	Pathogen/Plant Disease	Type of Plant	Mechanism	Reference
Dibasic and tribasic phosphate salts	*Colletotrichum lagenarium*	Cucumber	Influences the activity of apoplastic enzymes, such as polygalacturonases, thereby releasing elicitor-active oligogalacturonides from plant cell walls.	[78,79]
			Preceded by a rapid generation of superoxide and hydrogen peroxide.	
	*Blumeria graminis* f.sp. *hordei*	Barley	Reduces powdery mildew infection by 89%.	[80]
SA Derivatives	TMV	Tomato Tobacco	Establishes plant immunity by an accumulation of PR proteins.	[81]
Isonicotinic acid derivatives	TMV	Tobacco	Decreases the necrotic area on leaves.	[82]
	*Colletotrichum lagenarium*	Cucumber	Induces chitinase and modifies the physiology of the host.	[83]
Thiadiazole and isothiazole derivative	Powdery mildew, anthracnose, and bacterial leaf spot Alternaria leaf spot, anthracnose, bacterial shot hole	Wheat	Promotes the expression of defense-related genes and SA catabolism. Induces plant defense responses.	[84] [85]
		Pumpkin		
		Cucumber		
		Chinese cabbage		
		Strawberry		
		Peach		
β-Aminobutyric acid	*Alternaria brassicicola*, *Plectosphaerella cucumerina*	Arabidopsis	Promotes callose accumulation by an ABA-dependent defense pathway.	[86]

**Table 3:** Other examples of alginates that induce resistance against plant pathogens.

ALG Concentration	Pathogen	Plant	Mechanism	Reference
5 g/L	Tobacco mosaic virus (TMV)	Tobacco (on leaves)	The antiviral activity of ALG on infectivity of TMV on blocking the decapsulation process of TMV protein on the cell membrane surface.	[123]
50 g/L	*Botrytis cinerea*	Kiwifruit (on fruit)	Reduction in the incidence of gray mold and diameter of lesions of kiwifruit during storage; enhancing the activity of polyphenol oxidase, l-phenylalanine ammonia-lyase (PAL), and β-1,3-glucanase related to pathogen defense.	[124]
1 g/L	*Fusarium oxysporum* f.sp. *albedinis*	Date Palm (on roots)	The stimulation of PAL activity in roost; the increased transcriptional level; stimulates expression of the genes involved in phenolic metabolism and burst oxidation.	[125]
2 g/L	*Verticillium dahliae*	Olive (on twigs of 10 cm in length with 16 leaves)	Increase in the enzymatic activity of PAL in the stem; inhibitory rates on mycelial growth of the fungus in vitro.	[126]
0.3 g/L	*Erwinia carotovora* *Xanthomonas campestris*	soybean cotyledon	The accumulation of phytoalexin and inducing PAL in soybean cotyledon.	[127]
5 g/L AOS combined with *Meyerozyma guilliermondii*	*Penicillium expansum*	Pears (on Fruits)	Increase in the activities of superoxide dismutase (SOD), catalase (CAT), polyphenol oxidase (PPO), peroxidase. (POD), phenylalanine ammonia-lyase (PAL), chitinase (CHI), total phenol content, and flavonoid content in pears; reduce spore germination rate and inhibit the germ tube elongation of *P. expansum*.	[128]

## Data Availability

Not applicable.
